# Genome-wide association analysis reveals a novel QTL *CsPC1* for pericarp color in cucumber

**DOI:** 10.1186/s12864-022-08606-5

**Published:** 2022-05-19

**Authors:** Hongyu Huang, Qinqin Yang, Lidong Zhang, Weiliang Kong, Huizhe Wang, Aimin Wei, Shengli Du, Ruihuan Yang, Jiawang Li, Tao Lin, Xiaolin Geng, Yuhe Li

**Affiliations:** 1 State Key Laboratory of Vegetable Germplasm Innovation, Tianjin Kernel Cucumber Research Institute, Tianjin, 300192 China; 2 China Agricultural University College of Horticulture, Beijing, 100193 China; 3 Institute of Cucumber Research, Tianjin Academy of Agricultural Sciences, Tianjin, 300192 China

**Keywords:** Cucumber, Pericarp color, BSA-seq, GWAS, GATA transcription factor

## Abstract

**Background:**

Cucumber is an important melon crop in the world, with different pericarp colors. However, the candidate genes and the underlying genetic mechanism for such an important trait in cucumber are unknown. In this study, a locus controlling pericarp color was found on chromosome 3 of cucumber genome.

**Results:**

In this study, the light green inbred line G35 and the dark green inbred line Q51 were crossed to produce one F_2_ population. Consequently, we identified a major locus *CsPC1* (Pericarp color 1). Next, we mapped the *CsPC1* locus to a 94-kb region chromosome 3 which contains 15 genes. Among these genes, *Csa3G912920*, which encodes a GATA transcription factor, was expressed at a higher level in the pericarp of the NIL-1334 line (with light-green pericarp) than in that of the NIL-1325 line (with dark-green pericarp). This study provides a new allele for the improvement of cucumber pericarp color.

**Conclusion:**

A major QTL that controls pericarp color in cucumber, *CsPC1*, was identified in a 94-kb region that harbors the strong candidate gene *CsGATA1*.

**Supplementary Information:**

The online version contains supplementary material available at 10.1186/s12864-022-08606-5.

## Background

Pericarp color is a valuable trait in the horticulture industry because it strongly influences consumer preference and exhibits extensive phenotypic variation that can be used in breeding. Many quantitative trait loci (QTLs) and genes related to pericarp color have been detected and/or cloned in crops. In melons, pericarp color is determined by the pigments [1]. In muskmelon, an F-box coding gene *CmKFB*, was identified on chromosome 10 and functions as a post-transcriptional regulator [2]. *MEL03C003375* is an APRR2 gene in melon. The orthologous genes of MELO3C003375 in cucumber (Csa3G904140) [3], watermelon (ClCG09G012330) [4], pepper (GeneBank accession no. KC175445) [5] and tomato (SolyC08g077230) [5] have been demonstrated to control chlorophyll metabolism and pigment accumulation in pericarp [6]. And *MELO3C003097* [6], an ortholog of SG1 in Arabidopsis, is required for chloroplast development [7]. In watermelon, *qrc-c8-1* on chromosome 8 controls the green shade of pericarp; it was identified by high-density genetic mapping of recombinant inbred lines and explained 49.942% of the phenotypic variation in pericarp color [8]. *Cla002755* and *Cla002769* on chromosome 4 are markers for yellow pericarp and were identified by bulked segregant analysis sequencing (BSA-seq) and genome-wide association studies (GWAS) [9]. In a study of light and dark green pericarp in watermelon, a G → C mutation in the CLAPRR2 intron prematurely terminates variant transcript translation in light green watermelon [4]. Through fine mapping, CLCG08G01780 was a candidate gene associated with dark green rind and light green rind color in watermelon [10]. The wax gourd pericarp color gene (dark green vs. yellow) was first mapped to Chr5 based on the high-density genetic map [11]. *Bch05G003950* (*BhAPRR2*), encoding two-component response regulator-like protein *Arabidopsis* pseudo-response regulator2 (APRR2) was identified in a 179 kb region on Chr5, which is involved in the regulation of green and white pericarp color in wax gourd [12]. In tomato, *SlMYB12* was mapped to chromosome 1; it corresponded to the pink gene *y* and controlled the accumulation of yellow-colored flavonoids in the tomato fruit epidermis [13, 14]. In pepper, three independent pairs of genes (*y*, *c1*, and *c2*) and two QTLs (*pc8.1* and *pc10*) were identified as controlling ripe fruit color and chlorophyll content [15].

Cucumber (*Cucumis sativus* L., 2n = 2x = 14) is an economically important cucurbitaceous crop worldwide, with a total global production of 91.3 million tons, of which 72.8 million tons (79.7%) were produced throughout the Chinese mainland in 2020 (data available at http://www.fao.org/). The pericarp color of cucumber fruit is an important agronomic character that affects consumer choice. The locus *w* that controls the white pericarp of cucumber on chromosome 3 contains only one gene, *Csa3G904140* (*APRR2*) [16], which encodes a nuclear localization transcription factor and controls pericarp color by reducing the content of chlorophyll and chloroplasts [17, 18]. Cucumber *Csa7G051430* was identified by BSA-seq of extreme-phenotype F_2_ individuals from a cross between the light-green pericarp mutant *lgp* and the wild type 406. It is homologous to *Arabidopsis ARC5*, which plays an important role in chloroplast division [19, 20]. Similarly, *Csa6G133820*, mapped through the light-green leaf and pericarp mutant M218, encodes a Ycf54-like protein required for chlorophyll synthesis named *CsYcf54* [21, 22]. *Csa2G352940* (*CsMYB36*), encoding the transcription factor MYB36, regulates yellow-green peel color in cucumber [23]. To date, the mechanism that controls green pericarp color in cucumber remains unclear. Further study of pericarp color inheritance and identification of candidate genes associated with green pericarp color will therefore provide valuable information.

BSA-seq and GWAS are simple and effective methods for the identification of molecular markers associated with target genes and QTLs that control traits of interest [24, 25]. This study was designed to determine the inheritance pattern of green pericarp color and to map major pericarp color QTLs. BSA-seq analysis detected a genomic region harboring a major pericarp color QTL, *CsPC1*, on chromosome 3, and it was further validated by GWAS analysis. This study also provides preliminary evidence that *Csa3G912920* is the probable candidate gene in the *CsPC1* locus.

## Results

### Phenotypic analysis of pericarp color in cucumber

**Fig. 1:**
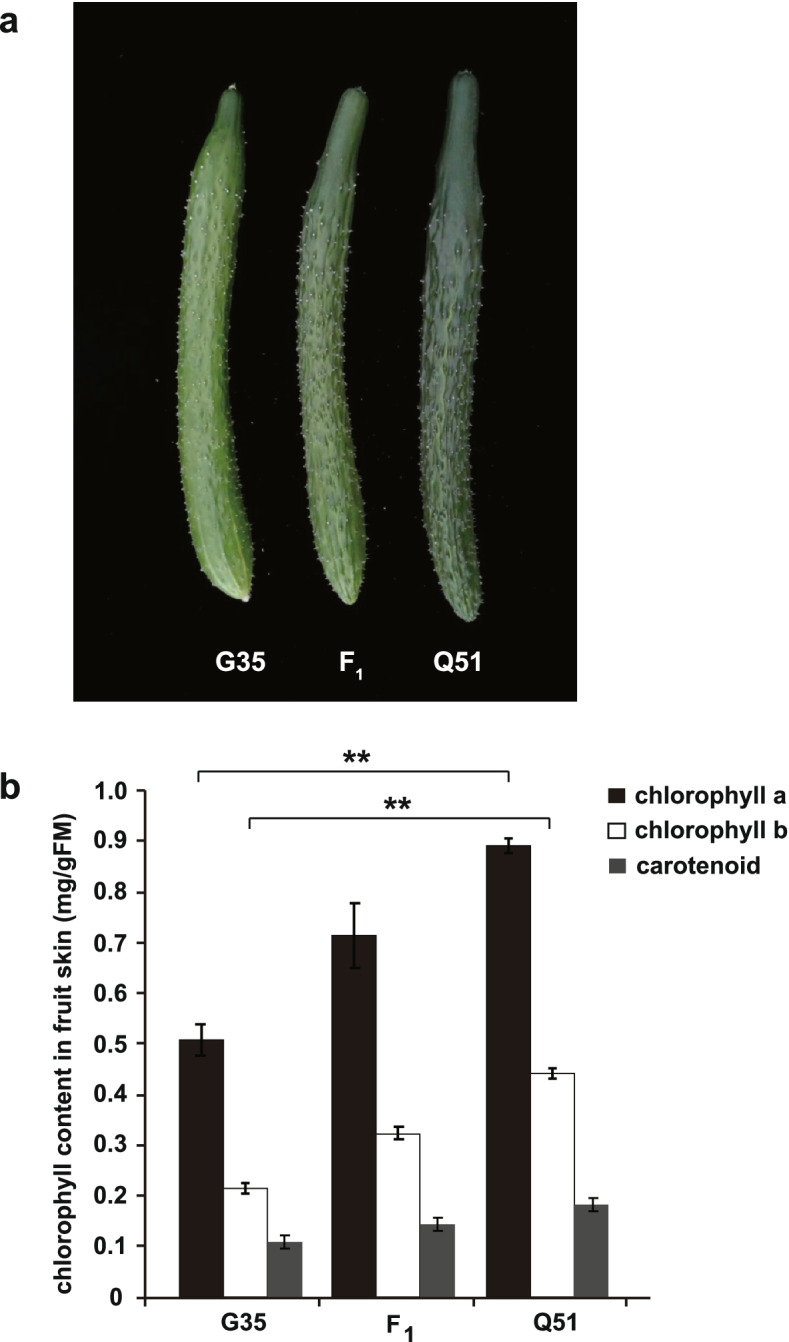
The pericarp color traits of two parents and their F_1_ hybrid. **a** G35 (P1, left), an F_1_ hybrid of G35 × Q51 (middle), and Q51 (P2, right). Photos of cucumber fruit were taken 10 days post-anthesis (DPA). **b** The content of chlorophyll a, b and carotenoid in two parents (G35 and Q51) and their F_1_ hybrid

The inbred lines G35 (light-green cucumber) and Q51 (dark-green cucumber) were used as parents for fine mapping of pericarp color. The pericarp color of all F_1_ individuals was darker green than G35 and lighter green than Q51, but it inclined more towards dark green (Fig. 1a). Pigment content analysis showed that chlorophyll a and chlorophyll b contents were significantly lower in G35 than in Q51. However, there was no significant difference in carotenoid content (Fig. 1b). These results indicated that green and light green pericarp color in the inbred lines G35 and Q51 was determined by chlorophyll content.

### Identification of a major QTL locus, CsPC1, on chromosome 3 by BSA-seq and GWAS

**Fig. 2:**
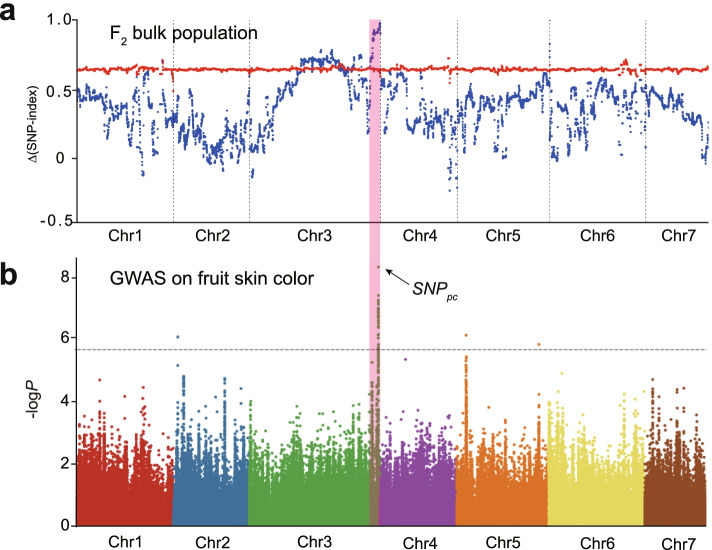
Identification of overlapping intervals identified by BSA-seq and GWAS for pericarp color in cucumber. **a** ∆(SNP-index) plot with statistical confidence intervals under the null hypothesis of no QTL (red, *P* < 0.01). The candidate QTL (*CsPC1*) location was identified between 36.62 and 39.77 Mb on chromosome 3. **b** GWAS analysis (Manhattan plots) showed a significant peak (*SNP*_*pc*_) above the threshold on chromosome 3 within the region previously identified in the QTL-seq analysis

To rapidly identify loci for pericarp color in the F_2_ population, two bulks consisting of 20 dark-green (SL-pool) and 20 light-green (QL-pool) progenies were sequenced on the Illumina platform. A total of 12.9 Gb of raw reads were generated, with an average depth of approximately 20.4 × . The short reads were aligned to the cucumber reference genome [26], and 145,804 SNPs were identified between the dark-green and light-green parents. Based on the SNP-indices of the QL- and SL-pools, the ∆(SNP-index) of a genomic region from 36.62 Mb to 39.77 Mb on chromosome 3 was greater than the threshold value and close to 1.00 (Fig. 2a). This region may therefore harbor a major QTL for the pericarp color trait in cucumber.

To independently confirm that this region was indeed related to pericarp color, GWAS was performed on 289 cucumber accessions (average depth of 19.73 × and 98.27% coverage of the cucumber reference genome) [26]. A total of 2,352,638 SNPs were identified using GATK software with default parameters [27]. To reduce the incidence of false-positive signals, a high-resolution variation map of 399,352 SNPs with minor allele frequency > 5% and missing rate < 0.2% was generated and used for genome-wide association analysis of pericarp color with a unified mixed linear model that controlled for population structure and familial relatedness. A Manhattan plot for cucumber pericarp color showed the strongest association signal (*SNP*_*pc*_) on the distal arm of chromosome 3, overlapping with the genomic region identified by QTL-seq (Fig. 2b). This indicated that a major QTL controlling pericarp color resided on the distal arm of chromosome 3, and it was named *CsPC1* (*Pericarp color 1*).

### Fine mapping narrowed down CsPC1 to a 94-kb interval

**Fig. 3:**
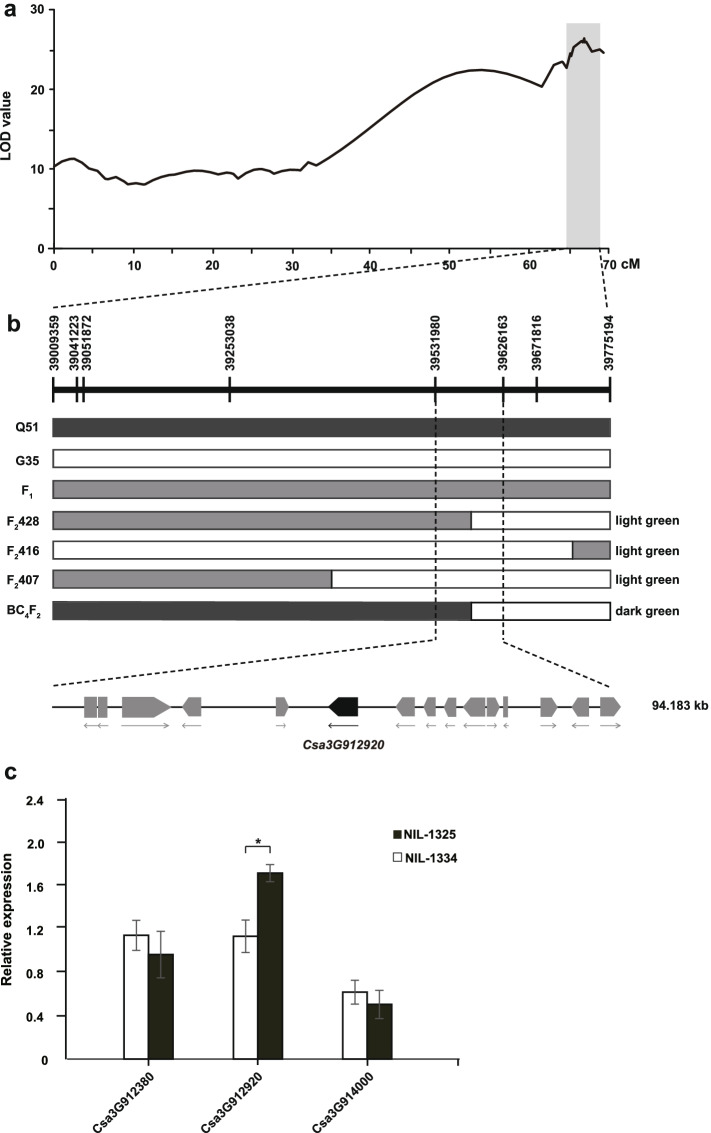
Fine mapping of *CsPC1* on chromosome 3*.* **a** LOD (log 10 of the odds ratio) plots of linkage analysis based on SNP markers indicate the most likely position of *CsPC1* between markers SNP39009359 and SNP39775194 on chromosome 3. **b** Mapping of the *CsPC1* region using three recombinants with extremely light-green pericarp color identified from 278 plants in the F_2_ and one BC_4_F_2_ individual. *CsPC1* was placed within a 94-kb interval containing 15 candidate genes between the markers SNP39531980 and SNP39626163. **c** Relative expression of three candidate genes in the fruit pericarp of the light-green near isogenic line NIL-1334 and the dark-green near isogenic line NIL-1325 at 0 days post-anthesis (DPA). The relative expression is shown as the mean ± standard deviation, and statistical significance was determined using Student’s *t*-tests (*P < 0.05)

To identify the candidate gene(s) in the *CsPC1* locus, classical QTL analysis was performed using 278 F_2_ progenies. A total of 35 SNP markers were developed between 15.66 and 39.77 Mb on chromosome 3 and used for genotypic analysis of the F_2_ segregating population (Supplementary Table S3). QTL analysis using an MQM showed that the LOD peak from 64.85 to 69.05 cM was consistent with the physical distance from 39.0 to 39.77 Mb on chromosome 3 (Fig. 3a). In this interval, the highest LOD marker explained 35.6% of the phenotypic variation in the F_2_ segregating population (Supplementary Table S1). The genomic interval of *CsPC1* was further narrowed down to between two SNP markers (39,531,980 and 39,626,163 bp) using four recombinant individuals from the F_2_ and BC_4_F_2_ populations (Fig. 3b). We therefore confirmed that the *CsPC1* locus lay within a 94-kb interval on chromosome 3.

### Identification of a candidate gene related to pericarp color

**Fig. 4:**
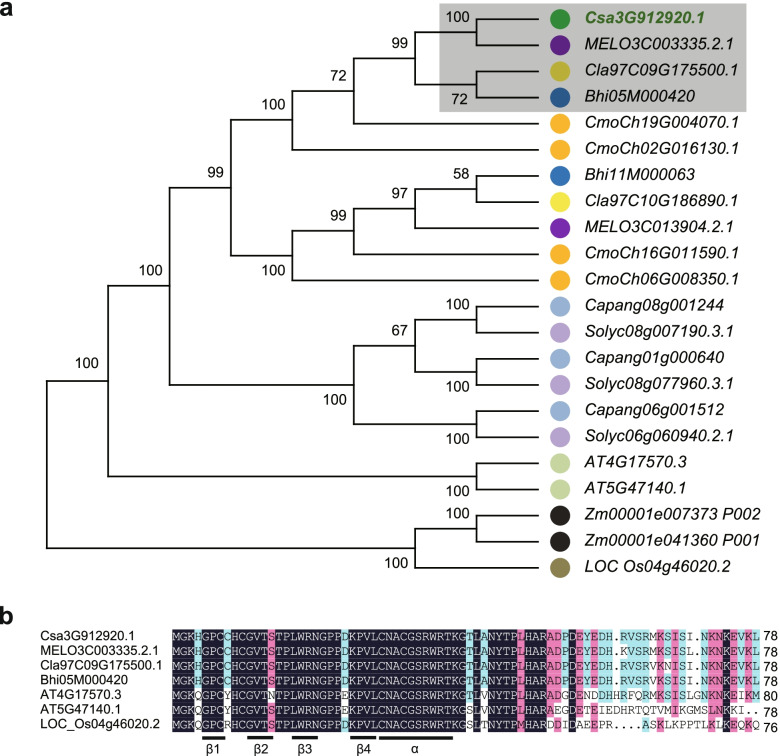
Phylogenetic tree and structure identity of *Csa3G912920* and its homologs in different species. **a** Phylogenetic tree of Csa3G912920 and its homologs in *Arabidopsis*, rice, maize, melon, watermelon, pumpkin, wax gourd, tomato, and pepper. The closest homologs of Csa3G912920 are indicated in a gray-shadowed box and include those from melon (MELO3C003335.2.1), watermelon (Cla97C09G175500.1), and wax gourd (Bhi05M000420). **b** Alignment of Csa3G912920, MELO3C003335.2.1, Cla97C09G175500.1, Bhi05M000420, AT4G17570.3, AT4G47140.1, and LOC_Os04g46020.2 protein sequences. Amino acid residues with at least 70.51% identity or similarity between these homologs are shaded black or red or blue, respectively

According to the cucumber genome database (http://www.icugi.org/), 12 of the 15 predicted protein-coding genes in the 94-kb interval have functional annotations (Supplementary Table S2). qPCR experiments were performed to investigate the expression patterns of three possible candidate genes associated with pericarp traits in NIL-1334 (light-green) and NIL-1325 (dark-green) (Supplementary Fig. S2). In the pericarp, only the expression of *Csa3G912920* differed significantly between NIL-1334 and NIL-1325 (*P* < 0.05) (Fig. 3c, Supplementary Fig. S3). The *Csa3G912920* gene encodes a plant GATA transcription factor and has a conserved zinc finger domain. A phylogenetic tree and sequence alignment showed that *Csa3G912920* homologs from melon (*MELO3C003335*), watermelon (*Cla97C09G175500*), and wax gourd (*Bhi05M000420*), highlighted in the gray-shadowed box, all encode GATA transcription factors (Fig. 4a and b). Secondary structural element analysis showed that the zinc finger domains include four β-folds and one α-spiral by looking up the literature (Fig. 4b). *Csa3G912920* was designated as a candidate gene for *CsPC1*.

Previous studies have shown that *Arabidopsis* GNC (GATA NITRATE-INDUCIBLE CARBON-METABOLISM-INVOLVED) and CGA1 (CYTOKININ-RESPONSIVE GATA1), members of the GATA transcription factor family, play a major role in the regulation of chlorophyll synthesis [28]. Under light, overexpression of GNC promotes chloroplast development and the production of chlorophyll in roots [29]. We therefore inferred that *Csa3G912920* is the probable candidate gene for *CsPC1* and named it *CsGATA1*.

## Discussion

In this study, we combined QTL-seq [30] of an F_2_ segregating population with GWAS to identify a major QTL *CsPC1* for pericarp color in cucumber. The main advantage of QTL-seq is that there is no need to develop DNA markers and marker genotyping. The SNP available between parental strains is such a marker, reducing cost and time. In addition, the use of SNP-index allows accurate assessment of the frequency of parental alleles. These advantages make QTL-seq an attractive method to quickly identify genomic regions containing major QTLs. However, each study is flawed. In this study, the GWAS data of one season is our deficiency. We recognize that repeating data over one more season is beneficial for drawing more robust conclusions, so we will pay more attention to this point in the future research.

Pericarp color is an essential agronomic trait in cucumber that affects exterior quality and consumer preferences. In this study, we detected the major QTL *CsPC1* on chromosome 3 between 39,531,980 and 39,626,163 bp. Previously, the *w* locus controlling the white pericarp trait was also mapped to chromosome 3 (Liu et al. 2016), residing 281 kb upstream of the *CsPC1* locus. In the *w* locus, *Csa3G904140* (*APRR2*) harbors a single-nucleotide insertion that causes a frameshift mutation and a truncated protein in the white cucumber. Here, we found no sequence differences in *APRR2* between the two parents, G35 and Q51. Therefore, *CsPC1* is a novel QTL that controls green pericarp in cucumber.

Through classical genetic mapping, *CsPC1* was narrowed to a 94-kb physical interval that contains 15 predicted protein-coding genes. The *Csa3G912920* gene encodes a GATA-type transcription factor, and its expression differed significantly between near isogenic lines with light- and dark-green pericarps. Previous studies have shown that the GATA transcription factor families are highly conserved in *Arabidopsis*, rice, and other plants [31]. The GATA transcription factors are evolutionarily conserved transcriptional regulators that recognize promoter elements with a G-A-T-A core sequence [32]. The paralogous LLM-domain B-GATA transcription factors GNC and GNL contribute to chlorophyll biosynthesis and chloroplast formation in light-grown *Arabidopsis* seedlings [28, 33, 34]. Together, GNC and GNL control germination, greening, flowering time, and senescence downstream of auxin, cytokinin, gibberellin, and light signaling [35]. Studies have confirmed that some GATA genes are preferentially expressed in the leaf [36]. Leaves are the main organs for photosynthesis and light stress response in plants. High expression of a GATA transcription factor in leaves is consistent with its influence on chlorophyll synthesis. Therefore, it is reasonable to suggest that *Csa3G912920* is the candidate gene for pericarp color in cucumber. Nonetheless, additional experiments are required to provide evidence for *Csa3G912920* gene function and robustly evaluate this hypothesis.

In conclusion, we identified a novel QTL, *CsPC1*, that controls green pericarp color in cucumber and proposed a candidate gene, *Csa3G912920*, that may be responsible for the green color phenotype. Our results provide insight into the biological and molecular mechanisms of pericarp color formation and can promote the development of attractive cucumber varieties with enhanced nutrients in the future.

## Materials and methods

### Plant materials and phenotype evaluation

Two cucumber inbred lines, G35 (light-green pericarp color) and Q51 (dark-green pericarp color), were crossed to create F_1_ progeny and then self-pollinated to generate an F_2_ population. The F_1_ progeny was backcrossed four times to the recurrent inbred parent G35 and then self-crossed to yield the BC_4_F_2_ generation. Chlorophyll a and chlorophyll b were extracted from pericarps of G35, Q51, and F_1_ progeny with ethyl alcohol and quantified by a spectrophotometric method. Two parental lines, together with the F_1_ and F_2_ generations, were used to describe and validate the inheritance pattern of pericarp color traits in immature fruit. Twenty F_2_ individuals with extremely light-green pericarp color and 20 with extremely dark-green pericarp color were selected for BSA-seq. Two hundred seventy-eight individuals from the F_2_ population were used for trait evaluation and QTL analysis (Supplementary Table S5). Pericarp color in the F_2_ population was independently evaluated by three persons. NIL-1334 (light-green pericarp) and NIL-1325 (dark-green pericarp) from the BC_4_F_2_ generation were used for gene expression analysis. Based on pericarp color, 289 cucumber accessions were classified into eight categories (white, yellow-white, white-green, yellow-green, light-green, green, dark-green, and black-green) (Supplementary Fig. S1) and used for GWAS analysis. The 289 cucumber GWAS accessions were grown in the plastic greenhouse of the Tianjin Kernel Cucumber Research Institute at the end of March 2017. According to the ecological type, there are 218 North China materials, 43 South China materials, 16 Japanese materials, and 12 European greenhouse materials, which are the main types of cucumbers in China. In mid to late June, three breeders with many years of breeding experience jointly investigated and graded the pericarp color of commercial melons. Each accession at least investigated three commodity melons.

### Genomic DNA and total RNA extraction

Genomic DNA was extracted by the cetyltrimethylammonium bromide (CTAB) method [37] from fresh young leaves of P_1_, P_2_, and F_2_ individuals and used for BSA-seq and QTL analyses.

Pericarp tissues were harvested from NIL-1334 and NIL-1325 at 0 days post-anthesis (DPA), 5 DPA, and 10 DPA. Each sample consisted of at least three fruits from different plants, and three replicate samples were used for gene expression analysis. Total RNA was extracted using the Quick RNA Isolation Kit (Huayueyang Biotechnology (Beijing) Co., Beijing, China) following the manufacturer’s instructions. The concentration of total RNA was quantified using a Nanodrop 2000 spectrophotometer (Thermo Fisher Scientific, Delaware, USA).

### BSA-seq

Two DNA pools, the light-green pool (QL-pool) and dark-green pool (SL-pool), were created by mixing equal amounts of DNA from 20 individuals with light-green pericarps and 20 individuals with dark-green pericarps, respectively. Paired-end sequencing libraries (150-bp read length) with insert sizes of approximately 400 bp were prepared for sequencing on the Illumina NovaSeq 6000 platform. The short reads from the two pools were aligned to the reference genome of the 9930 line [26] using BWA software with default parameters [38]. SNP-calling was performed using SAMtools and BCFtools [38]. Low-quality SNPs with base quality value < 30, read depth < 2 × , and mapping quality value < 30 were excluded to minimize false positives caused by repetitive genomic sequence or sequencing and alignment errors.

Two parameters, SNP-index and ∆(SNP-index) [30], were calculated to identify candidate regions for pericarp color QTLs. SNP-index is the proportion of reads covering a given SNP that differ from the reference sequence. Thus, SNP-index = 0 if all short reads covering a given nucleotide position contain the reference SNP (9930 line), whereas SNP-index = 1 if all the short reads at that position contain the mutant SNP. ∆(SNP-index) is obtained by subtracting the SNP-index of the QL-pool from that of the SL-pool. The average SNP-index at a given genomic interval was calculated using a sliding window with a 1-Mb window size and a 10-kb increment. SNP-index graphs for the QL-pool and SL-pool, as well as the corresponding ∆(SNP-index), were plotted. The ∆(SNP-index) should not differ significantly from 0 in a genomic region with no major QTL [30]. We used a R script simulation to generate confidence intervals around the SNP-index under the null hypothesis of no QTL. First, we created two pools of progeny with a given number of individuals by random sampling. From each pool, a given number of alleles were sampled, corresponding to the read depth. Second, the SNP-index for each pool and the Δ(SNP-index) were calculated, and the process was iterated 10,000 times for each read depth to generate confidence intervals. Finally, these intervals were plotted for all genomic regions with variable read depths.

#### GWAS

Re-sequencing data from 289 cucumber accessions (Supplementary Table S6) were obtained, with an average genome coverage of 98.27% and an average sequencing depth of 19.728 × . We obtained 2,352,638 SNPs, and 399,352 high-quality SNPs were retained, with a deletion rate of less than 0.2. The association between pericarp color and each SNP was tested using a unified mixed model [39, 40] that includes principal components [41] as a fixed effect to account for the population structure and kinship matrix [42] and to explain familial relatedness. Using the Bayesian information criterion, a backward elimination procedure was implemented to determine the optimal number of principal components to include in the mixed model [43]. The false discovery rate was controlled at 5% using the Benjamini and Hochberg procedure [44]. A likelihood ratio-based *r*^2^ statistic was used to assess the goodness-of-fit of each SNP [45]. All analyses were performed using the Genome Association and Prediction Integrated Tool (GAPIT) package [46].

### Marker development and QTL analysis

The SNPs were filtered from the re-sequencing data of the two parents, G35 and Q51. The sequence context of the candidate SNPs was examined in the 9930 reference genome using BLAST alignment to obtain longer sequences for marker development. In total, 35 kompetitive allele specific PCR (KASP) SNP markers on chromosome 3 were developed using the BSA-seq and GWAS data and created using Primer 5.0 (PREMIER Biosoft International, USA) (Supplementary Table S3). The genotypes of the F_2_ population were analyzed using an Infinite M1000 microplate reader (Tecan, Switzerland) and the online tool “snpdecoder” (http://www.snpway.com/snpdecoder/). Linkage analysis was performed using JoinMap 4.0 [47], and QTL analysis was performed in MapQTL6.0 using the multiple QTL model (MQM mapping) procedure [48](Van Ooijen, 2009).

### Quantitative real-time PCR (qRT-PCR)

Single-stranded cDNA was synthesized using the PrimeScript RT Reagent Kit with gDNA Eraser (TaKaRa Bio Inc., Dalian, China) following the manufacturer’s instructions. qRT-PCR was performed in a 10-μl reaction volume consisting of 5 μl TB Green Premix Ex Taq (Tli RNaseH Plus) (TaKaRa), 0.25 μl ROX Reference Dye (50 ×), 0.25 μl each of forward and reverse primers (10 μM), 1 μl cDNA templates, and 3.25 μl purified water. Thermal cycling began with an initial step at 95 °C for 30 s, followed by 40 cycles of 95 °C for 5 s and 60 °C for 34 s, and it was performed on the QuantStudio Flex 6 Real-Time PCR System (Applied Biosystems, California, USA). All samples were performed in triplicate, and *CsACTIN* (*Csa2G018090*) was used as the internal reference gene. Relative expression values were determined using the comparative Ct method (2^−ΔΔCt^). Primers used for qRT-PCR are listed in Supplementary Table S4.

### Phylogenetic analysis

*CsGATA1* and its homologous amino acid sequences were retrieved from public databases: SolGenomics (https://solgenomics.net/) and the Cucurbit Genomics Database (http://cucurbitgenomics.org). Known GATA transcription factors from rice, maize, and *Arabidopsis* were added to the analysis. Sequence alignments and a neighbor-joining tree with 1000 bootstrap replicates were constructed in MEGA X [49].

## Supplementary Information

**Additional file 1: Supplementary Figure S1. **Eight categories of pericarp colors were defined to evaluate phenotypes of 289 cucumber accessions. **a** White, **b** Yellow-white, **c** White-green, **d** Yellow-green, **e** Light-green, **f** Green, **g** Dark-green, **h** Black-green. **Supplementary Figure S2. **The phenotype of the light-green near isogenic line NIL-1334 and the dark-green near isogenic line NIL-1325. **Supplementary Figure S3. **Relative expression of three candidate genes in pericarp of the light-green near isogenic line NIL-1334 and the dark-green near isogenic line NIL-1325 at 0 day spost-anthesis (DPA), 5 DPA, and 10 DPA. The relative expression is shown as the mean ± standard deviation, and statistical significance was determined using Student’s *t*-tests (*P < 0.05). **Supplementary TableS1.** QTL analysis of pericarp color in the cucumber F_2_ population. **Supplementary Table S2.** Information on 15 candidate genes between 39,531,980 and 39,626,163 bpon chromosome 3. **Supplementary Table S3. **Information on 35 KASP SNP markers for QTL analysis. **Supplementary Table S4.** Primers used in qRT-PCR. **Supplementary Table S5. **The phenotype of the F_2_ population (278). **Supplementary Table S6. **The phenotype of the natural population used for GWAS (289). 

## Data Availability

The datasets supporting the conclusions of this article are included within the article and its additional files. The raw Illumina sequence reads have been deposited into the National Genomics Data Center (https://bigd.big.ac.cn/) under accession number CRA004282.
