# Supplementary material for: Genome-wide association analysis reveals a novel QTL CsPC1 for pericarp color in cucumber
Source: BMC Genomics. 2022 May 19;23:383. doi: 10.1186/s12864-022-08606-5 (PMC9121586; doi:10.1186/s12864-022-08606-5)
Supplement: Supplementary file 1 — Additional file 1: Supplementary Figure S1. Eight categories of pericarp colors were defined to evaluate phenotypes of 289 cucumber accessions. a White, b Yellow-white, c White-green, d Yellow-green, e Light-green, f Green, g Dark-green, h Black-green. Supplementary Figure S2. The phenotype of the light-green near isogenic line NIL-1334 and the dark-green near isogenic line NIL-1325. Supplementary Figure S3. Relative expression of three candidate genes in pericarp of the light-green near isogenic line NIL-1334 and the dark-green near isogenic line NIL-1325 at 0 day spost-anthesis (DPA), 5 DPA, and 10 DPA. The relative expression is shown as the mean ± standard deviation, and statistical significance was determined using Student’s t-tests (*P < 0.05). Supplementary TableS1. QTL analysis of pericarp color in the cucumber F2 population. Supplementary Table S2. Information on 15 candidate genes between 39,531,980 and 39,626,163 bpon chromosome 3. Supplementary Table S3. Information on 35 KASP SNP markers for QTL analysis. Supplementary Table S4. Primers used in qRT-PCR. Supplementary Table S5. The phenotype of the F2 population (278). Supplementary Table S6. The phenotype of the natural population used for GWAS (289). [file 12864_2022_8606_MOESM1_ESM.zip › 12864_2022_8606_MOESM1_ESM.docx]

**Genome-wide association analysis reveals a novel QTL *CsFS1* for fruit skin color in cucumber**

Hongyu Huang^1†^, Qinqin Yang^2†^, Lidong Zhang^1^, Weiliang Kong^1^, Huizhe Wang^3^, Aimin Wei^3^, Shengli Du^3^, Ruihuan Yang^3^, Jiawang Li^1^, Tao Lin^2^, Xiaolin Geng^2*^, Yuhe Li^3*^

🖂 Xiaolin Geng

gengxlwahg@163.com

🖂 Yuhe Li

liyuhe@126.com

1 State Key Laboratory of Vegetable Germplasm Innovation, Tianjin Kernel Cucumber Research Institute, Tianjin 300192, China

2 China Agricultural University College of Horticulture, Beijing 100193, China

3 Institute of Cucumber Research, Tianjin Academy of Agricultural Sciences, Tianjin 300192, China

^†^ Hongyu Huang and Qinqin Yang have contributed equally to this work.

* Corresponding authors

**Supplementary Information**

**Additional file 1: Supplementary Figure S1.** Eight categories of pericarp colors were defined to evaluate phenotypes of 289 cucumber accessions. **a** White, **b** Yellow-white, **c** White-green, **d** Yellow-green, **e** Light-green, **f** Green, **g** Dark-green, **h** Black-green.

**Additional file 2: Supplementary Figure S2.** The phenotype of the light-green near isogenic line NIL-1334 and the dark-green near isogenic line NIL-1325.

**Additional file 3: Supplementary Figure S3.** Relative expression of three candidate genes in pericarp of the light-green near isogenic line NIL-1334 and the dark-green near isogenic line NIL-1325 at 0 days post-anthesis (DPA), 5 DPA, and 10 DPA. The relative expression is shown as the mean ± standard deviation, and statistical significance was determined using Student’s *t*-tests (**P* < 0.05).

**Additional file 4: Supplementary Table S1.** QTL analysis of pericarp color in the cucumber F_2_ population.

**Additional file 5: Supplementary Table S2.** Information on 15 candidate genes between 39,531,980 and 39,626,163 bp on chromosome 3.

**Additional file 6: Supplementary Table S3.** Information on 35 KASP SNP markers for QTL analysis.

**Additional file 7: Supplementary Table S4.** Primers used in qRT-PCR.

**Additional file 8: Supplementary Table S5.** The phenotype of the F_2_ population (278).

**Additional file 9: Supplementary Table S6.** The phenotype of the natural population used for GWAS (289).
